# Formulation and Characterization of Bone-Targeting Vancomycin-Loaded Liposomes

**DOI:** 10.3390/pharmaceutics17060792

**Published:** 2025-06-18

**Authors:** Basel Karzoun, Wala’a Albenayan, Shilpa Raut, Eman Atef

**Affiliations:** 1 Department of Pharmaceutical Sciences, MCPHS-University, 179 Longwood Avenue, Boston, MA 02115, USA; baselkarzoun@hotmail.com (B.K.); walaa.albenayan@gmail.com (W.A.); 2 Cymbiotika LLC., 5825 Oberlin Dr, San Diego, CA 92121, USA; shilpa@cymbiotika.com; 3 School of Pharmacy, West Coast University, 590 N Vermont Ave, Los Angeles, CA 90005, USA

**Keywords:** vancomycin, liposomes, targeting, alendronate, bone, osteomyelitis

## Abstract

**Background:** We report the successful formulation of a bone-targeted vancomycin-loaded liposomal carrier. **Method:** The basic liposomal structure is composed of 1,2-distearoyl-sn-glycero-3-phosphocholine (DSPC), cholesterol, and dicetyl phosphate (DCP) in a molar ratio of 3:1:0.25, respectively. The dehydration–rehydration method was used to maximize the liposomal-encapsulation efficiency of vancomycin after the initial preparation using thin-film hydration. **Results:** Sodium alendronate was used as a targeting moiety and was successfully conjugated to DSPE–PEG–COOH via carbodiimide chemistry, as was confirmed using IR spectroscopy. The resulting conjugate, DSPE–PEG-alendronate, was subsequently used in the formulation of bone-targeting vancomycin-loaded liposomes. In vitro binding assays with hydroxyapatite demonstrated preferential binding of the surface-modified liposomes to hydroxyapatite crystals. Furthermore, ex vivo studies revealed that the surface-modified liposomes exhibited enhanced binding affinity to the tibial bone tissue of 4-week-old male CD1 mice, in comparison to unmodified liposomes. **Conclusions:** The successfully formulated surface-modified vancomycin loaded liposomes showed enhanced bone affinity with a great potential for targeting the antibiotic to infected bones.

## 1. Introduction

Osteomyelitis, an infection-induced inflammation of the bone, is predominantly caused by *Staphylococcus aureus*, including the methicillin-resistant strain (MRSA) [1,2]. Treating osteomyelitis effectively remains challenging due to poor antibiotic penetration into infected bone tissue and the emergence of resistant bacteria such as MRSA, which produce beta-lactamase enzymes that inactivate many conventional antibiotics [3,4]. It is evident that systemic antibiotics are the standard therapy in the treatment of osteomyelitis, but their effect is dependent on tissue penetration and blood supply. High-dose and long-term antibiotic therapy have further contributed to the emergence of resistant bacteria. Antibiotics that are commonly used against MRSA include ciprofloxacin, nafcillin, vancomycin, and third-generation cephalosporins, among others [5].

The application of local antibiotics has been used to overcome these drawbacks and has been used for both treatment and prophylaxis; approaches include but are not limited to the use of degradable or non-degradable antibiotic-loaded carriers.

Vancomycin is a glycopeptide antibiotic widely used as a first-line agent against MRSA infections. However, vancomycin presents several therapeutic challenges that limit its effectiveness in systemic therapy. Vancomycin exhibits poor penetration across biological membranes and compromised delivery to poorly vascularized bone tissues. This is mainly due to its high hydrophilicity, high water solubility, and limited lipid solubility. As a result, high doses and prolonged treatments are needed, and these increase the risks of nephrotoxicity and ototoxicity [6,7]. These limitations highlight the need for a targeting delivery system that can enhance vancomycin concentration specifically at the infected site in the bone, reducing systemic exposure and toxicity. This can be achieved systemically or locally. Locally applied vancomycin is delivered directly to the surgical site, resulting in much higher tissue concentrations compared to systemic administration, even if vasculature is compromised, with the additional advantage of minimizing systemic toxicity [8]. It has been used as treatment and prophylaxis for many years [9,10].

Liposomes are suitable delivery vehicles capable of encapsulating a highly water-soluble drugs [11]. These vehicles are ideal carriers for both hydrophobic drugs and hydrophilic drugs such as vancomycin. Water-soluble drugs are encapsulated within the core. Furthermore, liposomes can be modified to target specific organs, a major advantage of this type of delivery system. Their biocompatibility and membrane-mimicking composition reduce toxicity and immunogenicity. Selection of the appropriate lipid composition and preparation method is critical to optimizing drug loading, stability, and controlled release [12,13,14]. Successful loading of vancomycin requires lipids with high phase-transition temperatures (e.g., DSPC) that can provide rigid bilayers that minimize premature drug leakage [15]. DCP is a negatively charged lipid that helps stabilize the liposomes by creating a repulsive forces that reduces liposome aggregation. Additionally, it improves the drug loading of positively charged molecules. Since vancomycin is positively charged at pH 7, DCP can be used to help improve vancomycin loading [16]. Cholesterol is added for its well-known benefits to liposomal formulations. It stabilizes the liposomal membrane by inserting itself into the phospholipid tails. It also reduces leakage of water-soluble loaded drugs and promotes longer retention of loaded drugs. Lastly, PEGylated lipids allow for chemical conjugation with many targeting moieties [17]. Alendronate is a bisphosphonate molecule that has high affinity to hydroxyapatite, which is a major component of the bone. Its amine group allows conjugation with lipids without loss of its affinity for bone [18].

The method of liposome preparation can significantly impact the encapsulation efficiency for highly water-soluble drugs such as vancomycin. Conventional methods of liposome preparation often result in low encapsulation efficiency and premature drug leakage for highly water-soluble molecules. To overcome this, the dehydration–rehydration method has emerged as a promising technique that can substantially enhance the drug loading of such molecules [19].

The overall aim of our study is to develop surface-modified liposomes that target vancomycin to bone tissues and immobilize it there for potential use in the treatment of osteomyelitis.

## 2. Materials

Femur and tibia bones were purchased from Charles River Laboratory, MA, USA. Vancomycin hydrochloride was purchased from Letco Medicine, NW Decatur, Al. DSPE–PEG carboxylic acid and DSPC were purchased from Avanti Polar Lipids, Alabaster, AL, USA. Hydroxyapatite, N-hydroxysuccinimide (NHS), dicetyl phosphate (DCP), N,N-dicyclohexylcarbodiimide (DCC), cholesterol, sodium alendronate, dialysis tubing, and chloroform were purchased from Sigma-Aldrich CO., LLC., St. Louis, MO, USA. Other reagents and solvents, unless otherwise specified, were supplied by Mallinckrodh Baker Inc., Phillipsburg, NJ, USA. All other chemicals and reagents were of analytical grade.

### 2.1. Liposome Preparation

Liposomes were prepared using the thin-film hydration method, which was followed by the dehydration–rehydration method to improve encapsulation efficiency. Liposomes were composed of DSPC, cholesterol, and DCP in a molar ratio of 3:1:0.25, respectively.

The DCP and cholesterol were dissolved in a mixture of chloroform and methanol in a ratio of 9:1. The DSPC was provided in a chloroform solution. The liposomes’ final lipid concentration was 12 mg/mL (17.5 mM). Using a rotavap, a thin film was prepared in a round-bottom flask. The thin film was then hydrated with 5 mg/mL vancomycin in Sorensen phosphate buffer at pH = 7 using probe sonication. The 5 mg/mL dose was selected because it is the final concentration that is commercially administered to patients. The film was hydrated for 20 min, with a one-minute pause after each 10 min sonication. The hydration was carried out in an ice bath. Dehydration–rehydration vesicles were prepared from a thin-film-hydration liposome. The liposomes were flash frozen at −80 °C for five minutes, and the samples were placed in a lyophilizer overnight. The lyophilized liposomes were then rehydrated with 100 µL of deionized water (DI water) and were vortexed for one min. An additional 100 µL of DI water was added to the liposomes. The rehydration step is critical, as encapsulation efficiency decreases with increasing rehydration volume. The samples were again flash-frozen, and the dehydration–rehydration step was repeated [20].

### 2.2. Characterization of Liposomes

#### 2.2.1. Dynamic Light Scattering

The particle size, polydispersity, and zeta potential of liposomes were characterized by dynamic light scattering using a Brookhaven 90 plus size analyzer, Nashua, NH, USA. A 20 µL volume of liposomes was diluted with two mL of DI water before analysis.

The liposomes’ morphology was observed via transmission electron microscopy (TEM), JEM-1010-Jeol, Japan. The liposomes were adsorbed on a carbon grid and stained with phosphotungstic acid.

#### 2.2.2. Encapsulation Efficiency

An ultrafiltration method was used to determine encapsulation efficiency. The method was validated for reproducibility with variation in parameters such as centrifugation time, prefiltration dilution, and centrifugation force.

Ultrafiltration tubes with a molecular-weight cut-off of 10 kDa (Millipore, Billerica, MA, USA) were used.

One mL of the liposome formulation was diluted to 250 mL with Sorenson phosphate buffer. Then, a 0.5 mL diluted liposome suspension was placed in ultrafiltration tube. The tube was centrifuged at 4000 rpm for 5 min. The filtrate was analyzed using HPLC, and the encapsulation efficiency was calculated using the following equation:

$$\mathrm{EE}\%=\frac{\mathrm{Amount}\;\mathrm{of}\;\mathrm{drug}\;\mathrm{added}-\mathrm{Amount}\;\mathrm{of}\;\mathrm{unentrapped}\;\mathrm{drug}}{\mathrm{Amount}\;\mathrm{of}\;\mathrm{drug}\;\mathrm{added}}\times 100$$

### 2.3. Preparation and Characterization of DSPE–PEG-Alendronate

**Figure 1:**
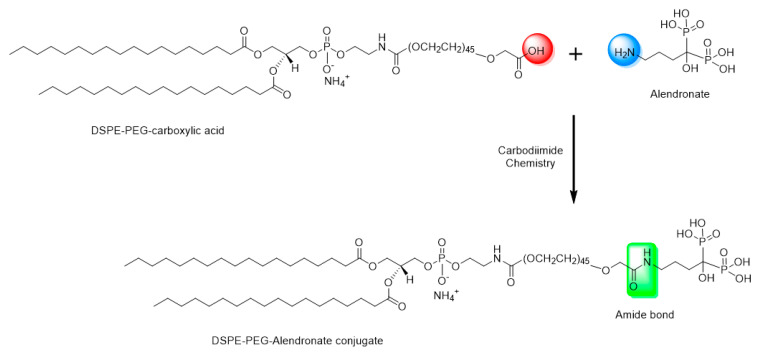
Schematic representation of the amide conjugation product (DSPE–PEG–alendronate). More details of the coupling process are given in Supplemental File S2.

Sodium alendronate has a free primary amine group. This theoretically makes conjugation with DSPE–PEG possible by formation of an amide bond between the carboxylic group of DSPE–PEG and the primary amine group of the alendronate (Figure 1).

DCC is a zero-length carboxyl-to-amine crosslinker, as no part of its chemical structure is incorporated into the final bond formed between the conjugated molecules. To prepare the DSPE–PEG-alendronate conjugate, as illustrated in Figure 1, 11.5 mg DSPE–PEG–COOH were dissolved in 2.5 mL acetone and activated with 2.1 mg N,N-dicyclohexylcarbodiimide DCC and 1.2 N-hydroxysuccinimide (NHS) overnight at room temperature. The insoluble byproduct, dicyclohexylurea, was removed using a 0.22 micron syringe filter. The activated lipid was then dried under nitrogen gas for two hours. One gram of sodium alendronate trihydrate and the activated lipid were dissolved in a 2.5 mL mixture of 2.25 mL DMSO and 0.25 DI water. The mixture was stirred for 24 h at room temperature. To remove the cross-linker, the cloudy mixture of the lipid conjugate and the cross-linker were placed in a benzoylated cellulose dialysis bag with a 2 kd molecular weight cutoff (Sigma-Aldrich CO. LLC., St. Louis, MO, USA). The bag was placed in 1000 mL DI water and stirred for 24 h at room temperature. The water was changed every six hours. The cloudy suspension was then taken out of the dialysis bag and centrifuged at 14,000 rpm for 10 min at 4 °C. The pellet and the supernatant were both dried under nitrogen gas for 24 h. The DSPE–PEG-alendronate conjugation product was dissolved in acetone, dried, and stored at −20 °C [21].

A thin-layer chromatography (TLC) analysis was carried out to check the final conjugation product. The mobile phase was composed of 65:25:4 *v*/*v*/*v* chloroform: methanol: water. The retention factor was calculated for both the final conjugation product and the unmodified DSPE–PEG–COOH.

To characterize the DSPE–PEG-alendronate conjugation product, IR spectroscopy analysis was used.

The IR spectra were analyzed to detect formation of amide bond between the carboxyl group of the DSPE–PEG and the amine group of the alendronate. The IR spectra of Physical mixture of DSPE–PEG and alendronate and the DSPE–PEG-alendronate conjugation product were collected using a Nicolet FT-IR spectrometer iS series (Thermo Scientific, Waltham, MA, USA). The spectra were compared and investigated for the presence of amide-specific peaks and shifts in the carbonyl peak of alendronate.

### 2.4. Hydroxyapatite Binding Study

The prepared conjugation was used to formulate the alendronate-surface modified liposomes by substituting 3% and 5% of the total volume of DSPC with DSPE–PEG–alendronate. In addition, 0.5% of the DSPC was substituted with rhodamine-PE. Hydroxyapatite (25 mg) was mixed with 250 µg of phosphate buffer in a 1.5 mL Eppendorf tube, and 25 µL of fluorescently labeled alendronate modified liposomes was added. The mixture was incubated for 1, 5, and 12 h. The tube was centrifuged, and the supernatant fluorescence intensity was measured using a plate reader (BioTek Synergy HTX Multimode Reader, Agilent, Temecula, CA, USA) at excitation/emission wavelengths of 530/590 nm. The binding percentage was calculated using the decrease in the intensity of the supernatant after incubation with hydroxyapatite via the following equation:

$$\mathrm{Binding}\;\mathrm{percentage}=\frac{x-y}{x}*100$$

where x is the fluorescence intensity of the initially fluorescently labeled liposomes without hydroxyapatite and y is the fluorescence intensity of the fluorescently labeled liposomes after mixing with hydroxyapatite.

### 2.5. Bone Binding Study

The tibias of four-week-old male CD1 mice (Charles River Laboratory) were incubated with 800 µL of Sorenson phosphate buffer (pH 7) and 200 µL of fluorescently labeled alendronate-surface-modified liposomes in 4 °C for five hours. The tibias were then washed with Sorenson phosphate buffer. The tibias were mounted on the microtome stage, and 20 micron slices were obtained using a sharp razor blade at −2 °C. The tissues were then visualized under a Nikon Eclips E600 fluorescence microscope using a TRITC cube filter. A 10× magnification lens and florescence mode were selected for imaging.

## 3. Results

The result indicated that the HPLC method developed in this study was linear over the range of 1–20 μg/mL. The method is linear in this range (R^2^ = 0.997). The limit of detection of the developed HPLC method is 1 µg/mL. The HPLC validation studies demonstrated both inter-and intra-day reproducibility, with relative standard deviations less than 10%. Further details are available in the Supplemental Materials (S1).

### 3.1. Liposome Characterization

**Figure 2:**
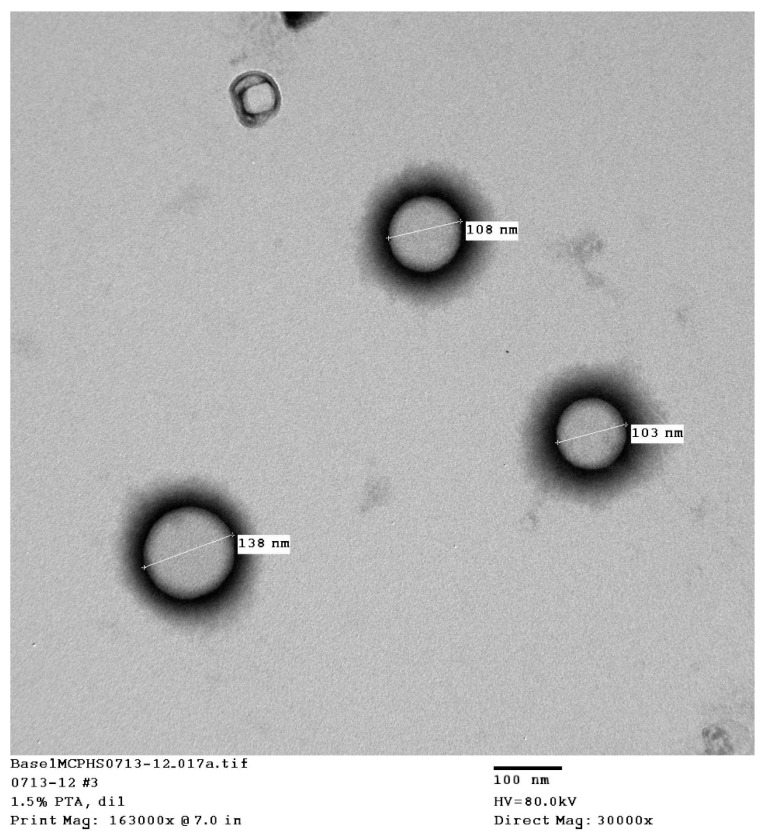
TEM images of vancomycin-loaded liposomes (DRVs).

The transmission electron microscopy (TEM) pictures showed that uniform, spherical, small, unilamellar liposomes were obtained (Figure 2). The results of characterization by dynamic light scattering (DLS) are presented in Table 1. The table summarizes the mean particle size, the polydispersity, and the zeta potential of the liposomes, which included liposomes formed from conjugated and unconjugated dehydration–rehydration vesicles (DRV) and those formed by thin-film hydration (TFH). All liposomes were composed of 3:1:0.25 DSPC:Ch:DCP.

The TFH liposomes were prepared using non–PEGylated lipids, which is expected to give the smallest particle size and the most homogeneous distribution via to the probe-sonication technique. The introduction of dehydration–rehydration (DRV) resulted in a slight increase in the particle size, since rehydration can result in larger liposomes due to the fusion of some particles. The addition of the alendronate-conjugated lipid increased the particle size of the liposomes. This was expected, since the pegylated and alendronate conjugated lipids are larger than the standard nonmodified lipids and will produce larger liposomes.

### 3.2. Determination of Encapsulation Efficiency

The encapsulation efficiency of a water-soluble drug is typically calculated by measuring the amount of unentrapped drug and subtracting that value from the total amount of drug added. The amount of unentrapped drug was measured using ultrafiltration. The two cycles of dehydration–rehydration increased the encapsulation efficiency to 21.9 ± 1.7% (*n* = 3).

### 3.3. Preparation of DSPE–PEG–Alendronate

**Figure 3:**
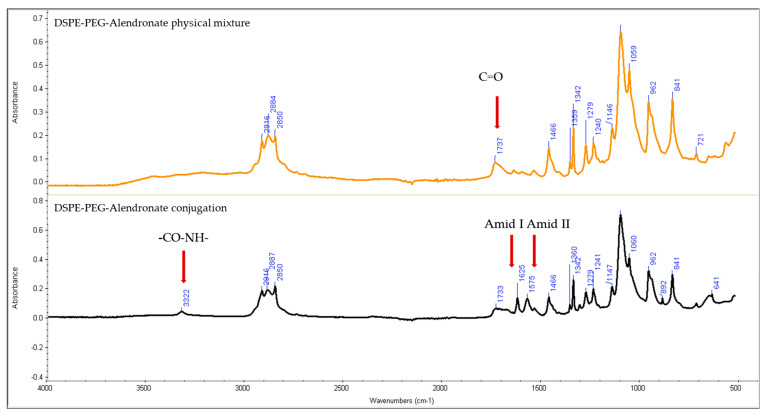
IR spectra of the physical mixture of DSPE–PEG and alendronate (**top**) and of the DSPE–PEG-alendronate conjugation product (**bottom**).

DSPE–PEG–alendronate conjugate was prepared as described in the Methods section and characterized using IR. Figure 3 shows the IR spectra of the physical mixture of DSPE–PEG and alendronate and of DSPE–PEG–alendronate.

Comparing the IR spectra of the physical mixture of DSPE–PEG and alendronate to that of the conjugation product, the later shows the characteristic sharp amide I and amide II peaks at 1625 and 1575 cm^−1^, respectively, suggesting the formation of an amide bond between the carboxyl group of the DSPE–PEG and the amine group of the alendronate. These peaks were not observed in the physical mixture of DSPE–PEG and alendronate. The amide I bond is a C=O group stretching vibration, whereas the amide II band is a combination of the N-H bending and C-N stretching vibrations of the group -CO-NH. A small secondary amide N-H stretching peak was observed at 3322 cm^−1^, indicating amide-bond formation. The formation of these amide bonds was accompanied by a decrease in the carboxylic acid stretching vibration of the C=O peak at 1737 cm^−1^. This peak was stronger in the physical mixture, confirming the change of environment of the carboxylic group (COOH) due to the formation of the amide bond and the successful conjugation of DSPE–PEG to alendronate.

### 3.4. TLC Analysis

The retention factor (Rf), defined as the ratio of the distance traveled by the compound to the distance traveled by the solvent front, was determined for both the final product (DSPE–PEG-alendronate) and the starting material (DSPE–PEG–COOH), yielding values of 0.64 ± 0.03 and 0.48 ± 0.03, respectively. These results indicate that the final product exhibits lower polarity compared to the starting material. Thin-layer chromatography (TLC) analysis also revealed the presence of impurities, as additional spots corresponding to reaction byproducts were observed. Based on the TLC data, purification of the conjugate via column chromatography is recommended to obtain a pure DSPE–PEG–alendronate product.

A figure illustrating the TLC analysis of DSPE–PEG–alendronate and DSPE–PEG–COOH is provided in the Supplemental File S3.

### 3.5. Hydroxyapatite-Binding Study of Surface-Modified Liposomes

**Figure 4:**
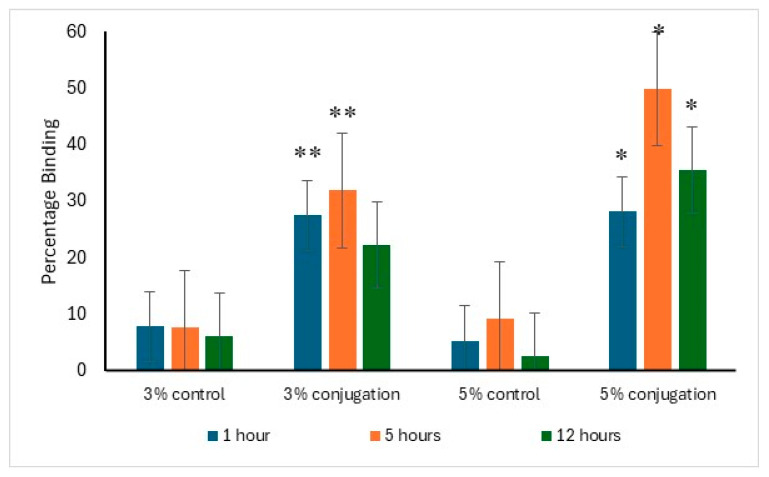
Percentage binding of alendronate-surface-modified liposomes to hydroxyapatite after 1, 5, and 12 h incubation (*n* = 3). * Represents significant increase in binding to hydroxyapatite of the 5% conjugation product compared to the control, *p* < 0.005. ** represents a significant increase in binding to hydroxyapatite of the 3% conjugation product compared to the control, *p* < 0.005.

The binding affinity of alendronate surface-modified liposomes to hydroxyapatite was evaluated in comparison to the control. After one hour of incubation, 27.5% and 28.2% of the liposomes with 3% and 5% DSPE–PEG-alendronate conjugation, respectively, exhibited binding to hydroxyapatite. The maximum binding was observed after five hours of incubation, with approximately 50% of the liposomes (5% conjugation) bound to hydroxyapatite, compared to 32% for the 3% conjugation. A decline in binding was detected after 12 h of incubation, likely due to detachment from hydroxyapatite caused by prolonged agitation. The data supporting this analysis are presented in Figure 4.

### 3.6. Bone-Binding Study of Surface-Modified Liposomes

**Figure 5:**
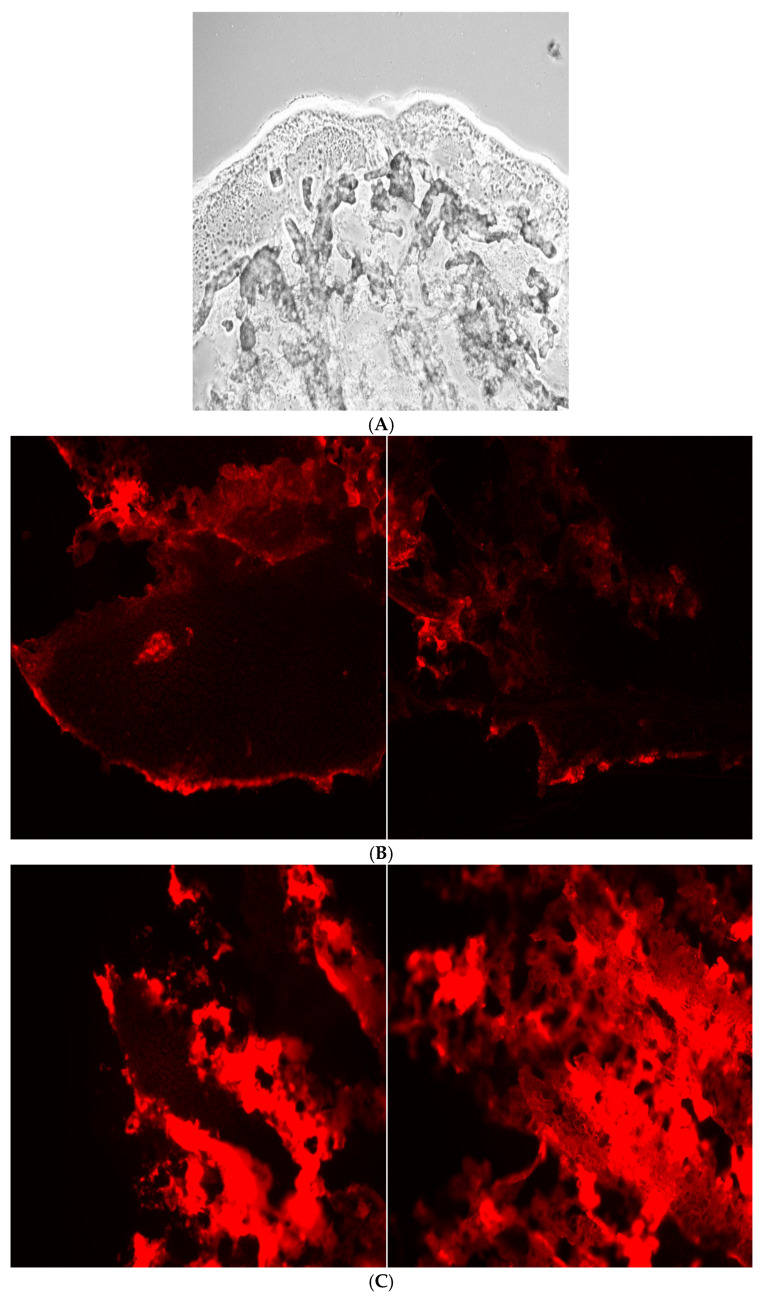
Fluorescence images of (**A**) negative control, (**B**) positive control, (**C**) 5% conjugation product.

The bone-binding study of surface-modified liposomes involved analysis of fluorescence images of the tibias after 5 h incubation with the alendronate-surface-modified liposomes, revealing more binding to hydroxyapatite compared to the positive control, as shown in Figure 5.

The tibia bone-binding study of surface-modified liposomes involved analysis of fluorescence imaging of the tibias following a 5 h incubation with alendronate surface modified liposomes. The results demonstrate an enhanced affinity in the modified, 5% conjugated liposomes compared to the positive control, as illustrated in Figure 5.

## 4. Discussion

Bisphosphonate has been used to target bone tissue. It enhances the local delivery of antibiotics to wounds. Buxton et al. investigated the therapeutic effects of ciprofloxacin conjugated to bisphosphonates, then incorporated into calcium phosphate granules. This approach differs from our targeting strategy, which involves modification of the lipid carrier rather than chemical alteration of the antibiotic molecule [22].

Our study demonstrates that incorporating vancomycin into surface-modified liposomes enhances its affinity for bone tissues, potentially facilitating drug immobilization and reducing the side effects typically associated with prolonged exposure to vancomycin in osteomyelitis treatment.

A few research groups have endeavored to incorporate vancomycin into liposomes in efforts to improve the drug’s pharmacokinetics [23,24,25,26].

Liposomes have been developed and investigated since 1965 to deliver and target small and large molecules. The surface of a liposome can also be modified to target drugs to the desired site of action. For all above reasons, liposomes were chosen as the drug carrier for vancomycin in this research. Several molecules with affinity for bones were suggested for use as bone-targeting moieties for vancomycin. Tetracycline, for example, binds strongly to bone and teeth [27,28], but its use as a bone-targeting moiety is limited because it must be in the correct orientation in order to bind to bone hydroxyapatite.

Sodium alendronate is a bisphosphonate molecule that is used to treat osteoporosis. It has a high affinity to hydroxyapatite, which is one of the major components of the bones. Its affinity comes from its two phosphonate groups, which bind to the calcium in the bone hydroxyapatite. When the amine group of the alendronate is conjugated with the carboxylic acid group of PEG, the molecule retains its binding affinity.

Reverse-phase evaporation and the dehydration–rehydration method of liposome preparation are normally used to maximize the encapsulation efficiency of water-soluble drugs. A very low encapsulation efficiency of vancomycin was achieved using the thin-film-hydration method. Upon two cycles of dehydration–rehydration, the encapsulation efficiency was increased to about 21.9%. When the solvent was removed by lyophilization, the lipids and the drug came into close contact with each other. After the hydration of the aggregated lipids, the lipids swelled, increasing the chance of entrapping the vancomycin. By repeating the dehydration–rehydration steps, we can maximize the encapsulation efficiency. The stability of vancomycin under these preparation conditions was validated using HPLC. The zeta potential was anticipated to be negative due to the presence of the negatively charged lipid DCP. The thin-film-hydration method, followed by dehydration–rehydration, resulted in the formation of small unilamellar vesicles, as confirmed by transmission electron microscopy. This is mainly due to the high amount of energy that was provided through the sonicator during preparation of the thin-film-hydration liposomes. To target the liposomes to bone tissue, alendronate was conjugated to DSPE–PEG–COOH using N,N-Dicyclohexylcarbodiimide (DCC) and N-hydroxysuccinimide (NHS). N,N-Dicyclohexylcarbodiimide (DCC) is a coupling agent that acts by activation of the carboxylic acid to form O-acylurea whichis the active intermediate product that reacts with amine-containing compounds, leading to amide-bond formation and precipitation of dicyclohexylurea in the reaction media. N-hydroxysuccinimide (NHS) is usually added with N,N-dicyclohexylcarbodiimide (DCC) to enhance the stability and prevent the epimerization of the intermediate product. The IR spectra, along with the TLC analysis, showed successful conjugation of the DSPE–PEG–COOH and alendronate. The sharp amide I and amide II peaks at 1625 and 1575 cm^−1^ provide strong evidence of the formation of an amide bond between the carboxyl group of the DSPE–PEG and the amine group of the alendronate. These peaks were not observed in the physical mixture of DSPE and alendronate. The decrease in the peak at 1737 cm^−1^ assigned to the carboxylic acid C=O stretching vibration was accompanied by the formation of these amide bonds. This carboxylic acid C=O stretching peak was stronger in the physical mixture and in the DSPE–PEG, confirming the formation of an amide bond.

The TLC analysis showed a difference in the polarity of the resultant conjugation product compared to that of the starting lipid. It also showed small secondary spots, suggesting that the filtration and the dialysis of the synthesis mixture was not sufficient to purify the conjugation product from the intermediate products, so further purification by column chromatography is recommended. The conjugation product was then used in the preparation of vancomycin-loaded liposomes by substituting 3% and 5% of the DSPC with the DSPE–PEG–alendronate conjugation product. To test the binding of the liposomes to bone tissue and hydroxyapatite, several methods have been used, including assessment of turbidity, lipid concentration, and fluorescence. The difference in the turbidity of the liposomes before and after mixing with hydroxyapatite could be used to determine the percentage of liposomes that bound to the hydroxyapatite crystals. This method is limited to hydroxyapatite crystal and cannot be used to assess ex vivo binding to bone tissue. The turbidity method is not an accurate method by which to assess binding. Another method that can be used to detect the binding to hydroxyapatite involves determination of the amount of lipids that bind to the hydroxyapatite crystals. This method is very accurate. However, it is time-consuming and complicated because it involves development of an HPLC method for the lipids used in preparing the liposomes. This method also lacks the ability to detect binding to bone tissue ex vivo. The most accurate method that can be used is the fluorescence method. This method can be used to detect in vitro and ex vivo binding. PE–rhodamine was used at 0.5% of the DSPC. The difference in the fluorescence intensity before and after the incubation with hydroxyapatite can give an accurate measurement of the binding to hydroxyapatite. This method also allows the visualization of bone tissue under a fluorescence microscope, which gives more valuable data about binding to the bone.

The data from the hydroxyapatite study correlated well with the bone-sectioning data.

Bone tissue is hard to section without decalcification, which can affect experimental accuracy due to alendronate binding to calcium in hydroxyapatite. To avoid this, tibias from 28-day-old male CD1 mice were used, as they were soft enough to be cut with a microtome [29].

Further studies need to be done to confirm bone targeting in vivo. The side effects associated with vancomycin therapy in mice need to be compared with those of the prepared formulation, especially in terms of ototoxicity and nephrotoxicity.

Toxicological studies of the long-term effects of alendronate are needed, and the amount of alendronate needed to exert the targeting effect should be determined.

## 5. Conclusions

Good encapsulation efficiency was achieved for vancomycin, a highly water-soluble drug, by the dehydration–rehydration method. The liposomal carrier was modified with alendronate, which was successfully conjugated to DSPE–PEG using DCC and NHS as coupling agents; conjugation was confirmed using TLC and IR. The alendronate-surface-modified liposomes showed significantly more binding to hydroxyapatite and bone tissue compared to the unmodified liposomes. While the in vitro and ex vivo data showed a promising result, the formulation should be tested in vivo in animal models to prove superiority over the standard treatment.

## Figures and Tables

**Table 1:** A summary of the mean particle size, polydispersity, and zeta potential of the liposomes formed from conjugated and unconjugated DRVs and those formed by TFH.

Liposomal Formulation	Mean Particle Size (nm)	Polydispersity	Zeta Potential (mV)
TFH	143.5 ± 3.8	0.160 ± 0.036	−53.9 ± 10.8
DRVs	155.9 ± 3.6	0.160 ± 0.039	−66.9 ± 3.2
TFH (conjugation)	171.6 ± 39.9	0.244 ± 0.058	−52.1 ± 9.7
DRVs (conjugation)	243.9 ± 28.6	0.299 ± 0.031	−47.36 ± 3.8

## Data Availability

The original data presented in the study are openly available in the main manuscript and the Supplemental Files.

## Supplementary Materials

The following supporting information can be downloaded at: https://www.mdpi.com/article/10.3390/pharmaceutics17060792/s1. File S1: High-performance liquid chromatography method development. Table S1. HPLC operating conditions for vancomycin hydrochloride. Figure S1. Vancomycin calibration curve (*n* = 3). File S2: Coupling mechanism of Alendronate-DSPCE-PEG. Figure S2. Coupling mechanism using DDC and NHS. File S3: TLC Analysis. Figure S3. TLC Analysis of: (1) DSPE-PEG-Alendronate, (2) DSPE-PEEG-COOH. Figure S4. Schematic representation of the proposed formulation.
