# Supplementary material for: Formulation and Characterization of Bone-Targeting Vancomycin-Loaded Liposomes
Source: Pharmaceutics. 2025 Jun 18;17(6):792. doi: 10.3390/pharmaceutics17060792 (PMC12196786; doi:10.3390/pharmaceutics17060792)
Supplement: Supplementary file 1 [file pharmaceutics-17-00792-s001.zip › pharmaceutics-3618304-supplementary.pdf]

Supplemental information:

S1: High-performance liquid chromatography method development:

A reverse-phase high-performance liquid chromatography (HPLC) method was developed. A survey scan between 200-400 nm was done to determine λ_{max} using ultraviolet spectrophotometer. The optimum chromatographic condition is summarize is table 9. The column used was ACE C18, 5µm, 250×4.6 mm. The mobile phase composition was KH2PO4 and acetonitrile in a ratio of 92:8 respectively. The KH_2PO_4 concentration was 20mM at pH=3.9. λ_{max} was determined to be 230 nm. The column temperature was 25 C^o. The injection volume was 20 µL and the flow rate was 1.2 ml/min. The retention time was found to be 11.4 min.

Table S1. HPLC operating conditions for vancomycin hydrochloride

Column	ACE C18, 5µm, 250×4.6 mm
Wavelength	230nm
Flow rate	1.2 ml/min
Retention time	11.4 min
Temperature	25 C^o
Injection volume	20 µL
Mobile phase	KH_2PO_4 0.02 M : ACN (92:8)

The limit of detection of this method is 1µg/ml. The method was linear in the range of 1-20µg/ml. A stock solution of vancomycin was prepared by dissolving 100 mg vancomycin hydrochloride in 100 ml DI water in a volumetric flask. This solution was used to prepare standard solutions of 1, 2, 5, 8, 10, 15, and 20µg/ml vancomycin solution. The method is linear in this range. The obtained equation was used in all of the later calculations.

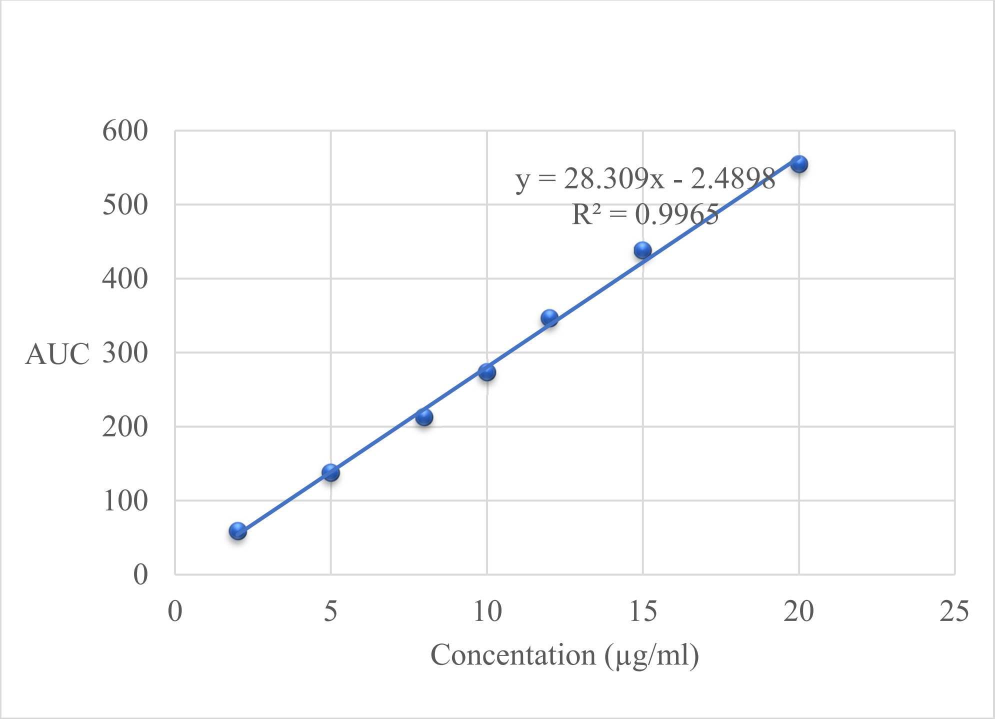

Figure S1. Vancomycin calibration curve (n=3)

S2: Coupling mechanism of Alendronate-DSPCE-PEG

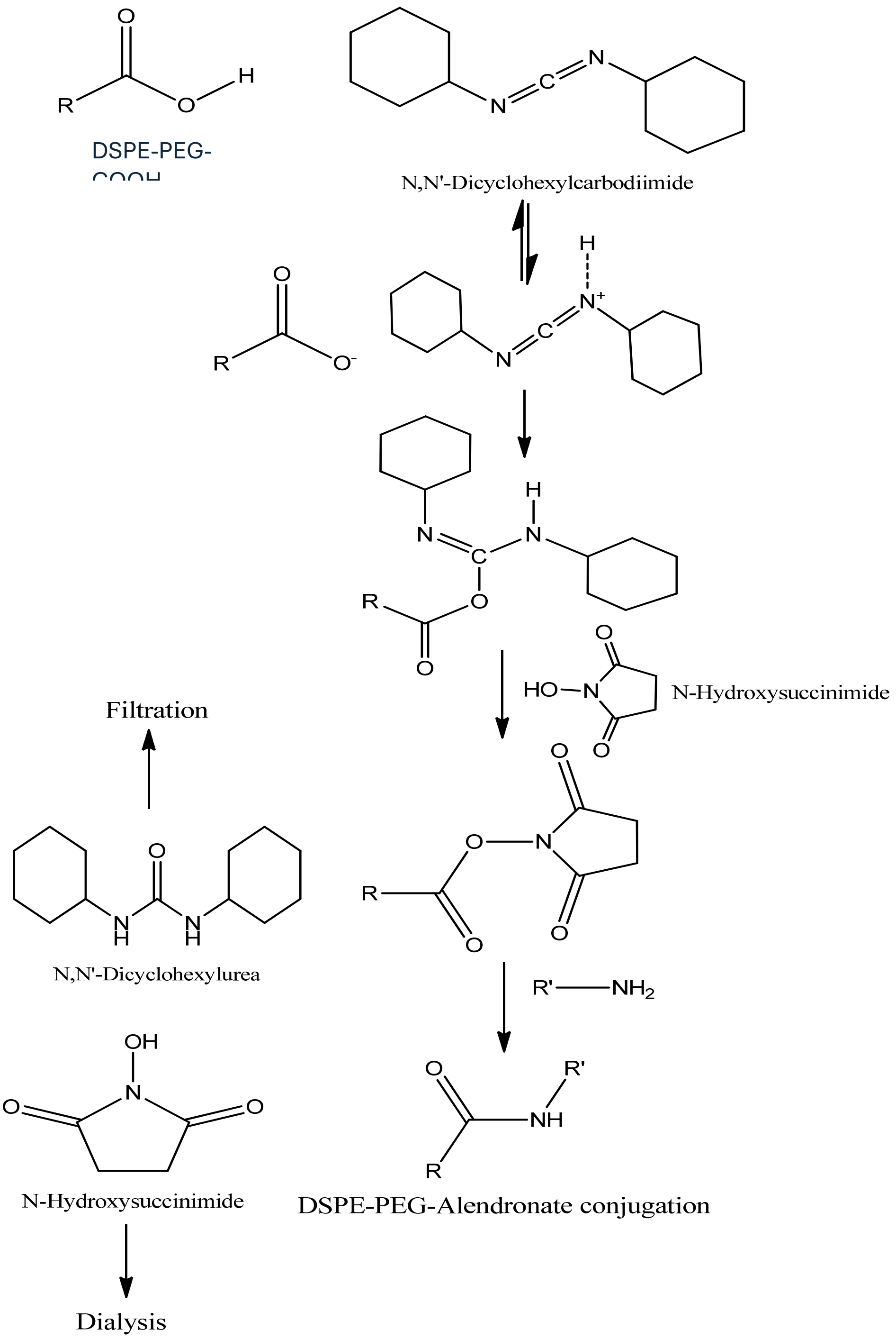

Figure S2. Coupling mechanism using DDC and NHS

S3: TLC Analysis:

The retention factor for the final product (DSPE-PEG-Alendronate) and the starting material (DSPE-PEG-COOH) was 0.64 ± 0.03, 0.48 ± 0.03 respectively. This suggests that the final product has less polarity than the initial starting material. The TLC analysis also shows that the final product was impure and spots for the reaction byproducts were shown in the TLC slide. The TLC data suggests that a purification of the conjugation using column chromatography is recommended to get a pure DSPE-PEG-Alendronate.

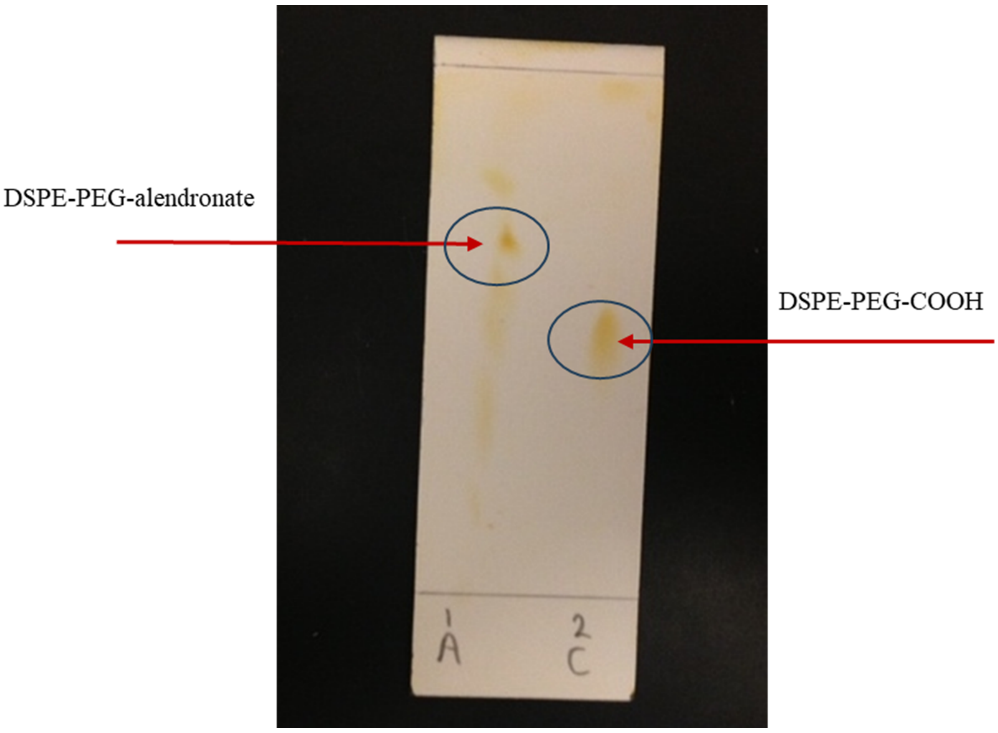

Figure S3. TLC Analysis of: 1) DSPE-PEG-Alendronate, 2) DSPE-PEEG-COOH

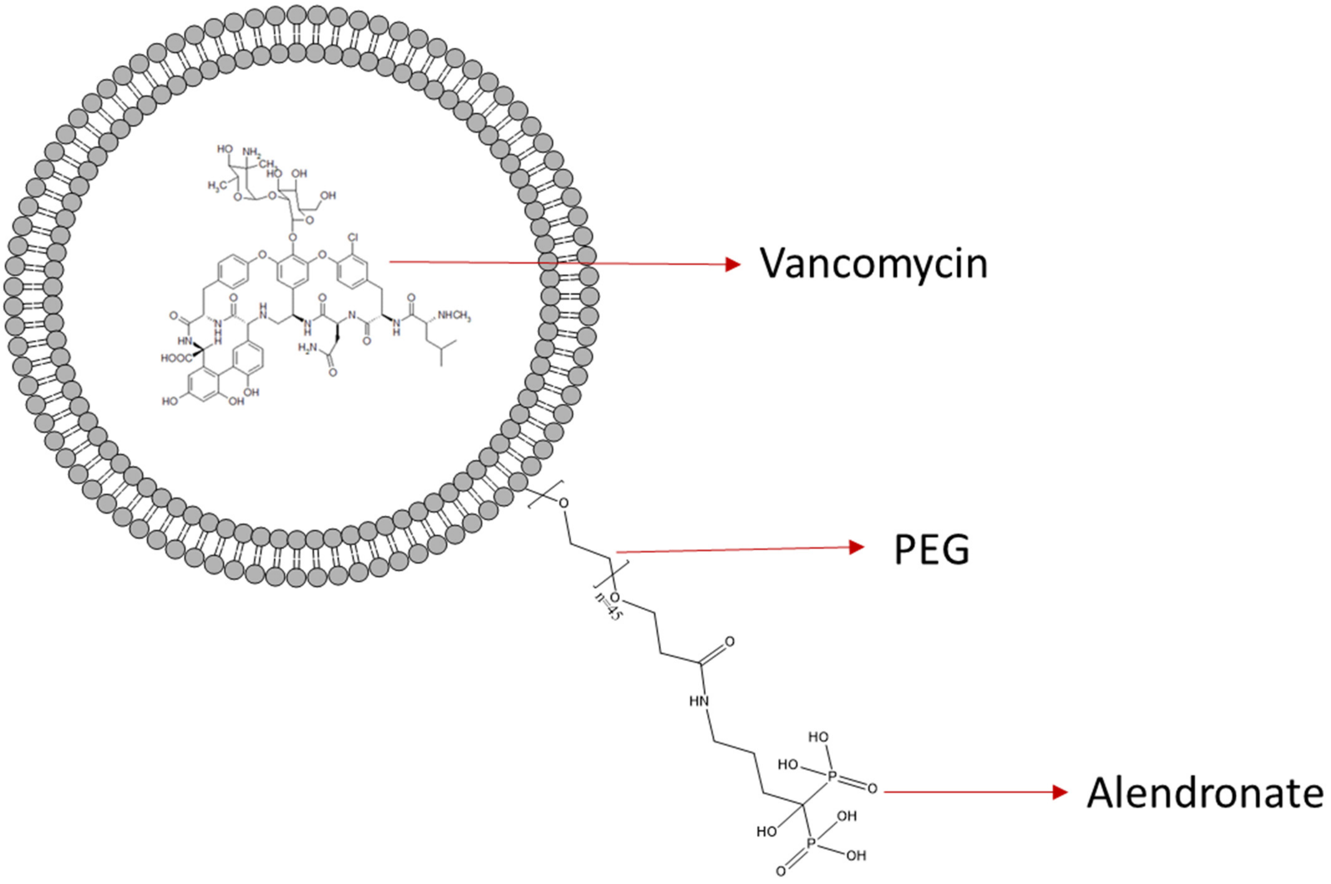

Figure S4. Schematic representation of the proposed formulation
